# Ologen Implantation versus Conjunctival Autograft Transplantation for Treatment of Pterygium

**DOI:** 10.1155/2018/1617520

**Published:** 2018-09-04

**Authors:** Xiuping Chen, Fei Yuan

**Affiliations:** Department of Ophthalmology, Zhongshan Hospital of Fudan University, Shanghai, China

## Abstract

**Purpose:**

To evaluate the effectiveness and safety of Ologen implantation versus conjunctival autograft transplantation for primary pterygium.

**Methods:**

A retrospective case-series analysis. Thirty-one eyes of 29 patients were included in the Ologen group and 42 eyes of 35 patients in the autograft group. The patients were followed up for 1 year and evaluated for slit-lamp biomicroscopy, intraocular pressure, and adverse events. Recurrence rate, complications, and final appearance of the cases were evaluated prospectively.

**Result:**

At 1 year after operation, 2 eyes recurred (6.5%) in the Ologen group and 4 eyes recurred (9.52%) in the autograft group. There was no statistically significant difference between both groups (*P*=0.157, *χ*^2^ = 3.781). There was no occurrence of serious complications. Two eyes among the 31 eyes of the Ologen group were conjunctivitis; the incidence of complications was 6.45% (2 eyes). There was conjunctivitis in 3 eyes of the autograft group, 1 eye complicated with symblepharon, and 1 eye with conjunctival granuloma; the incidence of complications was 11.90% (5 eyes), and there was no statistically significant difference between both groups (*P*=0.094). The conjuntiva was less vascular and inflamed at 1 month postoperatively in the Ologen group than in the autograft group.

**Conclusions:**

Ologen transplantation was technically easier, provided short operative time compared with conjunctival autograft transplantation, and preserved healthy conjunctiva with less complication and less recurrence; it may be a new, safe, and effective alternative for improving the short-term success rate of primary surgery.

## 1. Introduction

Pterygium, a wing-shaped fibrovascular proliferation growth that extends from the conjunctiva onto the cornea, is one of the most common conjunctival surface degenerative disorders [1]. Chronic ultraviolet light exposure, wind, dryness, and other factors may contribute to the formation of pterygium [2, 3]. It can impair vision through altered tear film [4], induced astigmatism, photophobia with a reported prevalence of 0.3% to 29% [3].

Currently, the only effective treatment for pterygia is surgery, but recurrence after surgery remains a great challenge. So, a number of different surgical approaches have been proposed which can be divided into 3 basic types: (1) bare sclera excision, (2) tissue grafting using conjunctival autograft, and (3) amniotic membrane or Ologen transplantation. Among these strategies, variable recurrence rates have been reported. The simple bare sclera excision is associated with a high recurrence rate up to 88% [5]. Conjunctival autograft transplantation showed low recurrence and complications rates [6]; it prevents recurrence by implanting the conjunctiva harvested from the superior bulbar conjunctiva to the bare sclera, acting as a barrier preventing migration of the nasal conjunctiva [7]. Although tissue grafting is significantly more time consuming and more difficult than leaving bare sclera, it shows a low recurrence rate from 5% to 15% [8]. However, these techniques are associated with their own complications [9].

Ologen (Ologen, Pro Top and Mediking Co./Ologen, Aeon Astron Europe, Netherlands), a 3D collagen-glycosaminoglycan scaffold, is specifically designed to promote wound healing with minimal scarring and low immunogenicity in a wide range of ophthalmic surgeries [10–12]. Our previous [13] study reported that Ologen, compared with mitomycin C for treatment of primary open-angle glaucoma, had a higher success rate and more efficacy bleb. It is considered as a safe, simple and effective alternative to the use of fibrosis disease. Herein, we report the efficacy of recurrence prevention-compared Ologen transplantation with conjunctival autograft surgery in primary pterygium.

## 2. Materials and Methods

A retrospective medical record review was conducted on patients who underwent pterygium surgery combined with Ologen or conjunctival autograft at Zhongshan Hospital of Fudan University between January 2006 and December 2015. All surgeries were performed by a single surgeon. A total of 136 patients (162 eyes) underwent both pterygium surgeries. Patients fulfilling the following study criteria were enrolled in the study: (a) diagnosis of a single-head primary pterygium on the basis of history and clinical examination; (b) a minimum follow-up period of 12 months; (c) age 18 years or more; and (d) no presence of coexistent ocular surface diseases. This study was conducted in accordance with the declaration of Helsinki and approved by the ethics committee of ZhongShan Hospital of Fudan University.

### 2.1. Surgical Methods

**Figure 1:**
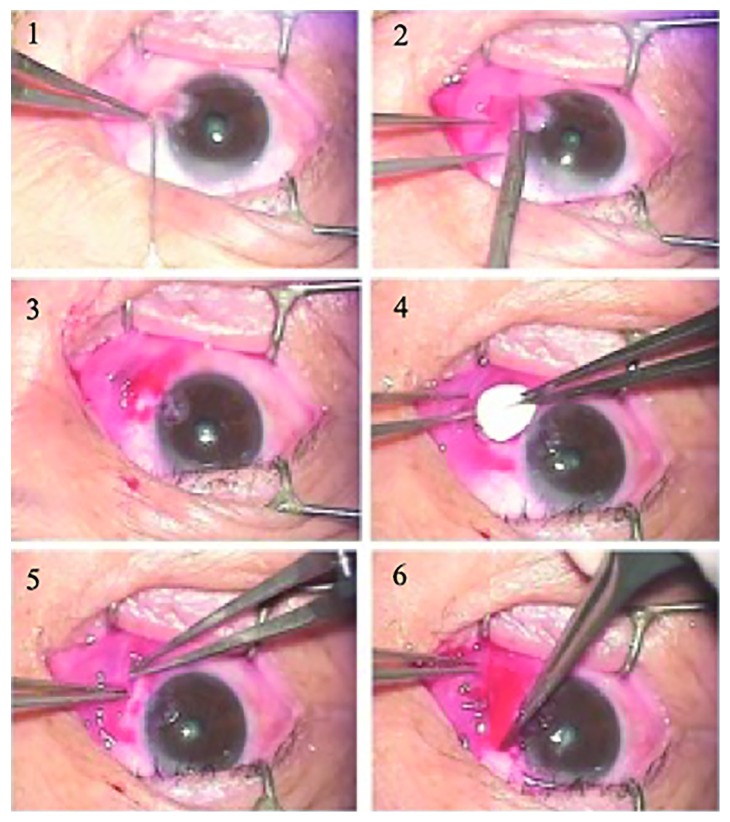
The surgery procedure of pterygium excision combined with Ologen implantation. 1: topical anaesthesia. 2: the head and body of the pterygium was excised by a scissor. 3: removed down to bare sclera. 4: put the Ologen under the conjunctival flap. 5: left a 2.0 mm width of bare sclera at the limbus. 6: sutured the flap with 10-0 nylon on the sclera.

In the Ologen group, all surgeries were performed by the same surgeon under a surgical microscope in the following manner and sequence: (1) topical anaesthesia was administered with 0.5% proparacaine; (2) the head and body of the pterygium which included the conjunctiva and underlying Tenon's layer was excised by a scissor; (3) removed down to bare sclera from the incision to the cornea limbus and dissected off the head with a surgical blade. The body of the pterygium which included the conjunctiva and underlying Tenon's layer was carefully excised; (4) after removing the remnant tissue on the sclera, put the Ologen under the conjunctival flap; (5) left a 2.0 mm width of bare sclera at the limbus; (6) sutured the flap with 10-0 nylon on the sclera (Figure 1). In conjunctival autograft transplantation group, a conjunctival free graft with similar size was obtained from the superotemporal bulbar conjunctiva. The autograft's limbal side was sutured to the cornea limbal side with 10-0 nylon. Operation did not exceed 30 minutes in all cases, and no complications occurred during surgeries.

Postoperatively, topical tobramycin 0.3% and dexamethasone 0.1% (Tobradex; Alcon Pharmaceuticals, Fort Worth, Texas, USA) was given every four times per day for 2 weeks. 10-0 nylon sutures were removed seven days after surgery. Patients were examined at 1 day, 1 week, 6 months, and 1 year postoperatively. During each visit, routine ocular examination was performed, including external eye photography, slit-lamp biomicroscopy, and intraocular pressure. Recurrence is commonly defined as a recurrent pterygium greater than 1 mm in size anterior to the limbus.

### 2.2. Statistical Analysis

Categorical variables were analyzed using the chi-square test, and continuous variables were analyzed using the unpaired Student's *t*-test. Continuous variables were presented as mean±standard deviation. Discrete variables were presented as percentages and analyzed using Fisher's exact test. The data were analyzed using SPSS software (version 21.0; SPSS Inc., Chicago, IL, USA). Correlation was considered significant at *P* value <0.05.

## 3. Results

The basic profile of patients in the study is shown in Table 1. Thirty-one eyes of 29 patients were included in the Ologen group and 42 eyes of 35 patients in autograft group. The mean age of the Ologen group patients was 56.73 ± 9.04 years (range 45–67 years) and 54.94 ± 10.52 years (range 42–73 years) in the autograft group. There were 10 (32.26%) males and 19 (61.29%) females in the Ologen group and 15 (35.71%) males and 20 (47.62%) females in the autograft group. Pterygium was diagnosed in 18 (58.06%) right eyes and 13 (41.94%) left eyes in the Ologen group. In the autograft group, it was diagnosed in 24 (57.14%) right eyes and 18 (42.86%) left eyes. The pterygium size in the Ologen group varied from 1.5 to 4.2 mm (mean 2.89 ± 0.64 mm). In the autograft group, it varied from 1.7 to 4.5 mm (mean 3.01 ± 0.52 mm). There was no statistically significant difference between both groups.

The postoperative recurrence rates are shown in Table 2. At 1 year after operation, 2 eyes recurred (6.5%) in the 31 eyes of the Ologen group, and 4 eyes recurred (9.52%) in the 41 eyes of the autograft group. There was no statistically significant difference between both groups (*P*=0.157, *χ*^2^ = 3.781).

The postoperative complications in both groups are shown in Table 3. Two eyes in 31 eyes of the Ologen group were conjunctivitis; the incidence of complications was 6.45% (2 eyes). There were conjunctivitis in 3 eyes of the autograft group, 1 eye concurring with symblepharon, and 1 eye complicated with conjunctival granuloma; the incidence of complications was 11.90% (5 eyes), and there was no statistically significant difference between both groups (*P*=0.094).

**Figure 2:**
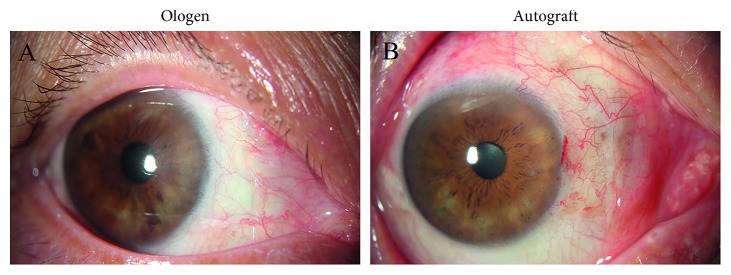
(a) Postoperative photo of Ologen group 1 month after surgery. (b) Postoperative photo of conjuntival autograft group 1 month after surgery.

Postoperative slit-lamp photographs are shown in Figure 2. The conjuntiva was less vascular and inflamed at 1 month postoperatively in the Ologen group than in the autograft group.

## 4. Discussion

Pterygium remains an important health care issue in China. The prevalence of pterygium was 3.7% in northern China and 37.4% in southern China [14], which suggested that ultraviolet exposure and ultraviolet radiation were important risk factors [15]. Up to now, surgery was the main treatment for pterygium [16]. Fibrosis causes adherence of the conjunctiva and Tenon's capsule, which may lead to recurrence, and it is dependent on the patient's inflammatory response [17]. The recurrence rate of simple excision results in high recurrence from 24% to 89%. The use of mitomycin C and other antiproliferative drugs can reduce the recurrence rate, but many complications such as delayed healing of the corneal epithelium and scleral ulcers can also be caused. Conjunctival autograft transplantation is a technically difficult and time-consuming procedure and a sacrificed part of the healthy conjunctiva. It may affect the outcome of surgical procedures that require healthy conjunctiva such as trabeculectomy if needed in the future for those patients [18].

Ologen is a three-dimensional porous collagen-glycosaminoglycan copolymer, taken from the matrix of porcine collagen, helps the reorganization of subconjunctival scar formation by separating the subconjunctival and episcleral tissues and inducing fibroblasts and myofibroblasts to grow in the structure after surgical excision of pterygium. It provides a good growth environment for the fibroblasts in the eye and guides the fibroblasts to grow on the pores of the matrix in a discrete manner, so that the wound's healing process will be physiological, and the tissue scar will be inhibited [19]. Remnants between the sclera and conjunctiva may induce recurrence, Ologen was used as a spacer to mechanically separate the subconjunctival and episcleral tissues to prevent fibrosis, it can completely fill this gap and may reduce the recurrence rate. The operation of Ologen implantation is simple.

In this study, Ologen was implanted under the conjunctiva at the same time while pterygium was excised. Two eyes in the 31 eyes were recurred at 1 year postoperatively. The recurrence rate was 6.45%. In the conjunctival autograft group, 4 eyes in the 42 eyes were recurred. The recurrence rate was 9.52%. There was no statistically significant difference between both groups (*P*=0.157, *χ*^2^ = 3.781). According to the results, the recurrence rate of pterygium after surgery was low in both groups. But in the Ologen group, the recurrence rate is lower than that of the conjunctival autograft group. The surgical complications in the Ologen group are two conjunctivitis; the incidence of complications was 6.45%. In the autograft group, there were three conjunctivitis, one symblepharon, and one granuloma. The incidence of complications was 11.90%. Though there was no statistically significant difference between both groups (*P*=0.094), in the Ologen group, the complications were less, the safety was higher. It is a safe and effective measure to reduce the postoperative recurrence and complication.

Postoperative inflammation caused by tissue damage induces activation of the fibroblasts of the subconjunctival tissue and induces recurrence [20]. According to the slit-lamp photographs taken 1 month postoperatively, the conjunctiva was less vascular and inflamed in the Ologen group than in the autograft group. This maybe attributed to Ologen's characteristics. Ologen is composed of a porous matrix of cross-linked atelocollagen and glycosaminoglycan. It contains thousands of microscopic pores and can induce fibroblast growth, leading to a minimal scarring and low immunogenicity healing process. Our previous study showed that Ologen created a diffuse bleb in trabeculectomy. It can inhibit scar formation postoperatively. Another important reason may be that Ologen implantation surgery is a time-saving and less tissue-damaging operation.

We concluded that both techniques used in the current study proved to be effective in reducing the recurrence rate after excision of primary pterygium with minimal complications in the short term postoperatively. Ologen implantation was technically easier, provided short operative time compared with conjunctival autograft transplantation, and preserved healthy conjunctiva. Ologen implantation has the advantage of less complication and less recurrence, it may be a new, safe, and effective alternative for improving the short-term success rate of primary surgery. We will continue to follow up and verify the effectiveness of Ologen. However, larger randomized trials are required to investigate the long-term efficacy and safety of this device.

## Figures and Tables

**Table 1:** Preoperative characteristics of patients in both groups.

Parameter	Ologen (*n*=31)	Autograft (*n*=42)	*P* value
Age (mean ± SD)	56.73 ± 9.04	54.94 ± 10.52	0.522^a^
Gender (male/female)	10/19	15/20	0.444^b^
Eye (right/left)	18/13	24/18	0.212^b^
Pterygium size	2.89 ± 0.64 mm	3.01 ± 0.52 mm	0.507^a^

^a^Independent Student's *t*-test. ^b^*χ*^2^ test or Fisher's exact test.

**Table 2:** The postoperative recurrence rates in both groups.

Group	Corneal invasion						Recurrence (1 y)
	<1/2 preoperation		Between 1/2 and 1		Equal to preoperation		
	6 m	1 y	6 m	1 y	6 m	1 y	
Ologen	1(3.23)	0(0)	0(0)	1(3.23)	1(3.23)	1(3.23)	2(6.45)
Autograft	1(2.38)	1(2.38)	0(0)	1(2.38)	1(2.38)	2(4.76)	4(9.52)
*P* value	0.626	0.524	N/A	0.626	N/A	0.431	0.157

**Table 3:** The postoperative complications in both groups.

Complications	Ologen	Autograft	*P* value
	(*n*=31, *n*(%))	(*n*=42, *n*(%))	
Conjunctivitis	2(6.45)	3(7.14)	0.668
Symblepharon	0(0)	1(2.38)	0.524
Granuloma	0(0)	1(2.38)	0.524
Total	2(6.45)	5(11.90)	0.094

## Data Availability

The original data used to support the findings of this study are available from the corresponding author upon request.
